# Boston Medical and Surgical Journal

**Published:** 1845-09

**Authors:** 


### BOSTON MEDICAL AND SURGICAL JOURNAL.

This Journal has entered upon the thirty-third volume, and is still improving with its advancing age. Its editor, J. V. C. Smith, M.D., is indefatigable. Nothing escapes him, and his weekly sheet has all the raciness and variety of a newspaper. We do not know a journal which keeps its readers so nearly up to all that is passing in the medical world. How adequately it is supported we have no means of knowing, but this we are persuaded of, that it merits a large share of patronage, and we hope is enjoying it. Y.

